# A Comparative Study on the Paradigm Shift in Golf Focusing on Participation Satisfaction, Switching Intention, Loyalty, and Continuous Participation Intention

**DOI:** 10.3390/bs16010114

**Published:** 2026-01-14

**Authors:** Mun-Gyu Jun, Chulhwan Choi

**Affiliations:** 1 Department of Professional Sports Coaching, Taegu Science University, 47, Yeongsong-ro, Buk-gu, Daegu 41453, Republic of Korea; junmg@tsu.ac.kr; 2 Department of Physical Education, Gachon University, 1342, Seongnam-daero, Sujeong-gu, Seongnam-si 13120, Republic of Korea

**Keywords:** golf, participation satisfaction, switching intention, loyalty, continuous participation intention

## Abstract

This study examines the recent diversification of the Korean golf market into traditional field, popular virtual reality (VR), and park golf, which is rapidly expanding among older adults. Comparing participants’ psychological characteristics and behavioral intentions across golf types is essential for sustainably developing the golf industry. Therefore, differences were investigated in participation satisfaction (physical, mental, and social), switching intention, loyalty, and continuous participation intention among regular participants in all three golf types in urban Korea. Data were analyzed from 327 adults aged 20 years or older (Field: 98, VR: 132, Park: 97) in Korea using on/offline surveys, and a multivariate analysis of variance with post hoc tests was implemented to compare psychological and behavioral differences across the three golf types. The findings showed that, first, physical and mental satisfaction were significantly higher in the park golf group than in the rest of the groups. Second, switching intention was higher in the field golf group than in the VR golf group. Third, loyalty and continuous participation intention were highest in the park golf group. Each golf type thus offers unique experiential value, with park golf particularly effective in fulfilling participants’ physical and psychological needs. Conversely, field golf faces potential risks of participant attrition because of cost and time burdens. The findings provide useful implications for predicting demand and developing differentiated marketing and management strategies tailored to generational needs.

## 1. Introduction

In modern society, the improvement of individual income levels and the expansion of leisure time have served as key social drivers promoting participation in various leisure sports (Choi et al., 2025; Jun & Choi, 2025). In the past, leisure activities were regarded as privileges limited to specific social classes; however, recent socioeconomic changes have accelerated the diffusion of sport participation as a more universal cultural phenomenon. Within this context, golf has evolved beyond being an exclusive sport for certain groups and has instead established itself as a popular leisure activity enjoyed by a wide range of age groups, including both middle-aged adults and younger generations (Bailey et al., 2019). In particular, the global COVID-19 pandemic provided an unexpected opportunity for growth in the Korean golf industry (S.-Y. Kim, 2024). As social distancing and restrictions on indoor sports facilities limited many forms of physical activity, the demand for golf surged dramatically, as it was perceived as a relatively safe outdoor sport (Yun & Seo, 2024).

At the center of this phenomenon is the emergence of a new group of consumers—the MZ generation (Kaczor, 2021). As digital natives familiar with technology and media environments and driven by a strong desire for self-expression, they actively shared their golf activities on social media (SNS), thereby creating a new cultural dimension of golf participation. This online-centered participation culture transformed golf into a means of self-branding and, consequently, stimulated the explosive growth of the domestic golf market (Johns et al., 2023). According to statistics from the Korea Golf Association (2021), golf participants in Korea increased from 4.7 million in 2019 to 5.64 million in 2021, with approximately 1.15 million belonging to the MZ generation. This large-scale influx of new participants has been closely linked to the growth of a new technology-integrated form of golf, namely virtual reality (VR) golf, also known as screen golf.

As of 2024, 9568 VR golf centers were operating nationwide in Korea, enabling participants to overcome traditional barriers such as time, cost, and weather, thereby providing an accessible environment for all (Bennett et al., 2025). Such accessibility significantly lowered the entry threshold for golf participation, and VR golf has emerged as a major participation space utilized by approximately 45.4% of all golf participants (Korea Golf Association, 2021). This technological innovation has accelerated changes in participation styles and consumption behavior across the leisure sports industry and has demonstrated that technological advancement functions as a central axis in restructuring the sports industry (D.-K. Kim et al., 2024; Lee & Kim, 2024a, 2024b).

Meanwhile, the rapid aging of Korean society has generated another type of demand for golf (S.-H. Kim et al., 2025). As older adults increasingly pursue leisure activities that promote both health and social relationships, park golf—a sport characterized by low physical burden, simple rules, and low economic cost—has emerged as an ideal alternative (S.-H. Kim et al., 2025). According to data from the Korea Park Golf Association, the number of registered members increased dramatically from approximately 16,000 in 2017 to approximately 106,000 in 2022, representing a 537% rise in just five years (Yeo & Kwon, 2024). This surge clearly demonstrates that park golf has evolved beyond a mere recreational activity for older adults and has become a social platform that promotes both health and community bonding. Consequently, the domestic golf market has undergone a structural transformation, becoming distinctly segmented into three categories: traditional field golf, VR golf led by the MZ generation, and park golf favored by senior participants.

However, the social and environmental changes before and after the COVID-19 outbreak have led to a paradigm shift in the overall leisure sports market (Choi et al., 2023; D.-K. Kim et al., 2023a). Recently, the enthusiasm of the MZ generation toward golf has rapidly declined, with many participants shifting their interest to other sports such as tennis and running (S.-Y. Kim, 2024). This shift is attributed to multiple factors, including high participation costs, long time commitments, and slow skill improvement relative to effort and investment (Yun & Seo, 2024). According to a recent report, the number of MZ-generation golf participants has decreased by more than 60% (Portugal et al., 2020), suggesting that the once-booming golf market is now facing both excessive competition and potential attrition of its core consumer base.

Under these changing market conditions, there is a growing need for an in-depth analysis of behavioral patterns and psychological characteristics of participants in different golf types (field, VR, and park golf) across generations. Specifically, identifying the factors that lead certain age groups to remain in their current golf type (loyalty and continuous participation intention) or to shift to another type (switching intention) constitutes a crucial task for developing sustainable strategies in the golf industry (Heo, 2024). Previous studies have predominantly focused on single themes or participant groups—such as service quality perceptions among VR golf participants (S.-Y. Kim, 2024), participation motivation among park golf participants (S.-H. Kim et al., 2025), and golf consumption culture among the MZ generation (Yun & Seo, 2024). In contrast, few studies have comprehensively examined the entire golf market divided into field, VR, and park golf while comparing intergenerational differences and potential transitions among participant types.

By classifying golf participants by generation and activity type, this study empirically evaluates the structural differences in their psychological and behavioral responses. The findings offer a broader understanding of consumer transitions within the diversifying golf market, providing a baseline for sustainable industry growth. Ultimately, this research assists in navigating the paradigm shift in the golf industry by offering insights into cross-generational demand and long-term retention strategies. The psychological factors examined in this study are not independent variables but are interconnected through a cognitive-behavioral process (Ajzen, 1991; Oliver, 1999). Participation satisfaction serves as the primary cognitive appraisal of the sporting environment, which significantly shapes the affective commitment and loyalty of participants (Casper & Stellino, 2008; Zeithaml et al., 1996). In the context of the rapidly diversifying Korean golf market, these psychological states act as critical determinants of whether a participant remains in a specific segment or seeks alternatives, defined as switching intention (Bansal & Taylor, 1999). By analyzing these factors within a unified framework, this study elucidates the complex decision-making processes of recreational golfers across different technological and environmental settings.

## 2. Conceptual Framework and Hypotheses

**Figure 1:**
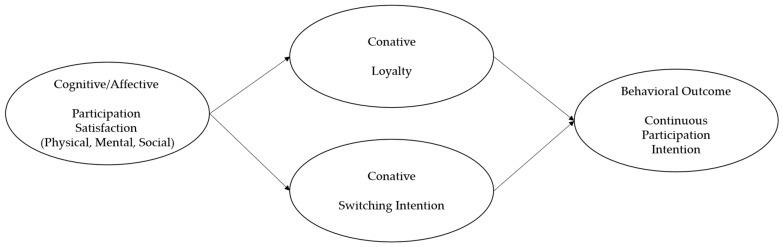
Conceptual Framework.

The primary objective of this study is to empirically compare the psychological and behavioral characteristics of participants in field, VR, and park golf. The conceptual framework is grounded in the cognitive/affective–conative hierarchy of effects (Figure 1). Within this framework, participation satisfaction, representing cognitive and affective evaluations, functions as a critical antecedent that simultaneously influences loyalty and reduces switching intention at the conative stage, ultimately shaping continuous participation intention as the behavioral outcome.

Prior studies in sport management have consistently demonstrated that the quality of leisure experiences and the resulting satisfaction are key determinants of long-term retention and loyalty. While traditional field golf has long served as the benchmark form of participation, the emergence of technology-based VR golf and senior-oriented park golf introduces distinct environmental and psychological stimuli. Theoretically, the present study integrates these diverse participation contexts into a unified analytical framework to examine how the satisfaction–loyalty–retention linkage is differentially manifested across participation segments.

From a practical perspective, identifying these mechanisms enables sport managers to move beyond generalized marketing approaches and develop segment-specific retention strategies. By situating the analysis within an established theoretical sequence, this study offers a rigorously articulated scientific foundation rather than ad hoc observations. Based on this conceptual framework and prior empirical evidence, the following hypotheses were formulated.

**H1.**  *Significant differences exist in participation satisfaction among field, virtual, and park golf.*

**H2.**  *Significant differences exist in switching intention among field, virtual, and park golf.*

**H3.**  *Significant differences exist in loyalty among field, virtual, and park golf.*

**H4.**  *Significant differences exist in continuous participation intentions among field, virtual, and park golf*.

## 3. Materials and Methods

### 3.1. Data Collection Procedure

The target population comprised adults aged 20 years or older who were regularly engaged in at least one of the three golf types within the Republic of Korea. Both online and offline survey questionnaires were utilized to ensure broader accessibility and minimize potential sampling bias. Each questionnaire included a clear statement outlining the purpose of the research, ethical considerations, and detailed instructions for participation. The respondents were encouraged to complete the survey at their own convenience, with assurances that their responses would remain anonymous and be used solely for academic purposes.

Data collection was conducted over a three-month period beginning on 26 July 2025. During this period, researchers distributed and retrieved questionnaires through golf facilities. A quantitative research design was employed, and data were obtained using a non-probability convenience sampling method commonly used in behavioral and sports science research emphasizing accessibility and voluntary participation. All participants were informed of the study’s objectives and procedures prior to participation, and their involvement was entirely voluntary, reflecting the ethical principles of informed consent and participant autonomy. A total of 500 questionnaires were distributed, of which 350 were collected. After excluding 23 inconsistent or incomplete responses, 327 valid questionnaires were utilized for the final analysis.

### 3.2. Instrument

First of all, sociodemographic information (e.g., gender, age, golf experience, and participation time per week) was utilized from the survey respondents. Additionally, the type of golf they have regularly participated in was used as an independent variable for the comparative analysis in this study.

Next, the constructs used to measure participation satisfaction and continuous participation intention among the survey respondents were based on the questionnaires modified by Choi et al. (2024), who examined consumer behaviors toward physical activity in exergaming environments utilizing VR. The participation satisfaction construct comprised three distinct sub-dimensions: (a) physical satisfaction (three items), (b) mental satisfaction (three items), and (c) social satisfaction (three items), reflecting the multidimensional nature of satisfaction experienced during sport participation. Meanwhile, continuous participation intention was conceptualized as a single-scale factor construct comprising three items designed to assess individuals’ willingness to persist in their current golf activities over time.

Furthermore, the construct used to capture the participants’ switching intention, defined as their likelihood of transitioning from one sport to another, was derived from a questionnaire modified by Im (2021), who explored the behavioral intentions of tennis participants. The switching intention construct was treated as a single-scale factor and contained three items that specifically measured the potential tendency to change sport participation types under certain motivational or situational conditions.

Finally, the loyalty construct, representing the participants’ attachment and commitment to a specific type of golf, was measured using items adapted from Min and Woo (2023), who investigated behavioral patterns among golf participants. The loyalty construct was operationalized as a single-scale factor comprising four items, reflecting attitudinal and behavioral loyalty toward continued engagement in the same golf activity.

All questionnaire items were measured using a five-point Likert scale ranging from 1 (“not at all”) to 5 (“very much”), where higher scores indicated stronger agreement or greater intensity of the respective psychological construct. This standardized measurement approach facilitated the reliability and comparability of responses across different participant groups and constructs.

### 3.3. Scale Validity and Reliability

To ensure the structural integrity of the measurement model, a confirmatory factor analysis (CFA) was conducted using the maximum likelihood estimation method (Table 1). This approach was employed to verify the adequacy of previously validated scales within the specific context of the Korean golf market diversification, as recommended by Hair et al. (2019). The model fit indices indicated a good fit between the proposed measurement model and the observed data (χ^2^/df = 1.95, CFI = 0.942, TLI = 0.931, RMSEA = 0.058, and SRMR = 0.045). All indices satisfied commonly accepted criteria, with CFI and TLI values exceeding 0.90 and RMSEA below 0.08, confirming the adequacy of the measurement model.

Convergent validity and internal consistency were assessed using composite reliability (CR), average variance extracted (AVE), and Cronbach’s alpha. All Cronbach’s alpha coefficients exceeded the recommended threshold of 0.80. CR values ranged from 0.815 to 0.882, surpassing the 0.70 criterion, while AVE values ranged from 0.528 to 0.715, exceeding the 0.50 threshold suggested by Hair et al. (2019), thereby indicating strong convergent validity. Discriminant validity was further supported, as the square root of the AVE for each construct was greater than the corresponding inter-construct correlations.

### 3.4. Data Analysis

The collected survey data were statistically processed and analyzed using IBM SPSS Statistics version 28.0. Initially, descriptive statistics were computed to summarize the participants’ sociodemographic characteristics and provide an overview of the sample distribution across the three golf participation types. Frequency and percentage analyses were conducted for categorical variables, thereby offering a foundational understanding of the dataset prior to inferential testing.

Subsequently, the validity of the measurement scales was examined through exploratory factor analysis (EFA) to confirm the construct’s underlying structure. The reliability of the scales was then evaluated using Cronbach’s alpha coefficients for six key factors: participation satisfaction (comprising physical, mental, and social sub-factors), switching intention, loyalty, and continuous participation intention. These procedures ensured that all measurement instruments demonstrated adequate internal consistency and construct validity—essential prerequisites for subsequent statistical comparisons.

Finally, a multivariate analysis of variance (MANOVA), followed by post hoc tests, was performed to identify statistically significant differences in the dependent variables among the three groups categorized by the type of golf activity (field, virtual, and park golf). MANOVA was deemed appropriate as it allows for the simultaneous testing of multiple dependent variables and helps control for Type I errors that could arise from conducting multiple univariate analyses. This analytical approach has been widely employed in previous social science studies (Jun & Choi, 2025; Yang et al., 2024; Choi et al., 2023, 2024; D.-K. Kim et al., 2023a, 2023b) that sought to compare mean differences in psychological or behavioral constructs across groups defined by categorical independent variables.

## 4. Results

### 4.1. Survey Respondents

Based on the collected golf activity information, the survey respondents were segmented into three groups: field golf participants (Field), VR golf participants (VR), and park golf participants (Park). Detailed sociodemographic information of the survey respondents is presented in Table 2.

### 4.2. MANOVA

A MANOVA was implemented to test differences in participation satisfaction (physical, mental, and social), switching intention, loyalty, and continuous participation intention. First, the homogeneity of covariance was verified (Box’s M = 81.548, F = 1.889, *p* < 0.01). In addition, statistically significant differences among the three types of golf groups (field, virtual, and park golf) were found (Wilks’ Lambda = 0.763, F = 7.715, *p* < 0.01, partial η^2^ = 0.127). In particular, the statistical analysis revealed significant mean differences in five dependent variables: (a) physical participation satisfaction, (b) mental participation satisfaction, (c) switching intention, (d) loyalty, and (e) continuous participation intention.

To determine which golf groups showed statistically significant mean differences, post hoc analyses were performed. First, in terms of physical participation satisfaction, the park golf group had relatively higher scores than the VR golf group. Next, the mental participation satisfaction, loyalty, and continuous participation intention, the park golf group had relatively higher scores than the field golf group and the VR golf group. Finally, regarding switching intention, higher average scores were obtained for the field golf group than the other two groups (VR golf group and park golf group). Detailed results of the MANOVA (Table 3) and post hoc analyses (Table 4) are reported below.

## 5. Discussion

The empirical results of this study demonstrate that each golf type offers unique experiential value to participants, as evidenced by significant group differences across all variables except social satisfaction. These findings suggest that while the social nature of golf remains consistent across environments, the physical and psychological benefits—along with behavioral intentions—are distinctly segmented by participation type. A detailed interpretation of these findings is provided below.

First, physical satisfaction was found to be significantly higher in the park golf group than in the VR golf group. This indicates that the physical characteristics of outdoor park golf, in contrast to the limitations of indoor VR golf, positively influenced the participants’ physical satisfaction. In particular, park golf involves low physical burden (S.-H. Kim et al., 2025) and enables health promotion within a pleasant natural environment (Bond et al., 2021), demonstrating its positive impact on maintaining physical health among older adults whose activity levels tend to decline with age. Furthermore, as park golf participants are generally older than those in field golf, the sport’s moderate level of physical activity may have contributed to satisfaction not merely through exercise intensity but also by providing an optimal level of bodily engagement.

Second, psychological satisfaction was also significantly higher in the park golf group than in both the field and VR golf groups. As the number of park golf participants has surged, particularly among older adults (Yeo & Kwon, 2024), this result implies that park golf plays an important role not only in promoting physical health but also in enhancing participants’ psychological and emotional well-being. In an aging society where depression and social isolation among older adults have emerged as major issues (Cacioppo & Cacioppo, 2014), participation in park golf appears to provide psychological benefits such as a sense of accomplishment, stress relief, and emotional stability, all of which can contribute to improving quality of life. Moreover, the novelty of participation in park golf may have provided higher levels of immersion compared with field or VR golf, which the participants may have experienced for longer periods.

Third, no significant group differences were observed in social satisfaction. This finding can be interpreted as resulting from the common social nature of all three golf types—field, VR, and park—each of which inherently involves companionship and interaction. Regardless of the type or setting of the activity, participants generally experience similar levels of social bonding and satisfaction through golf as a medium of social exchange. Thus, rather than suggesting that one particular sport yields higher social satisfaction, these results highlight that participation in any form of leisure sport that encourages interpersonal relationships leads to positive social outcomes.

Fourth, switching intention was significantly higher among the field golf participants than among those in the VR golf group. This finding empirically supports the phenomenon discussed in the introduction, namely, the post-COVID decline in golf participation (S.-Y. Kim, 2024). The high costs and time requirements associated with field golf (Yun & Seo, 2024) appear to act as persistent barriers. Recent economic instability and rising leisure expenses have likely increased the financial burden on field golfers, prompting some to discontinue their participation or shift toward other sports such as tennis or running. These findings suggest that different types of golf, which share the same origin but vary in participation context, attract participants by fulfilling diverse needs through their distinct appeals. Therefore, this result should be interpreted not as a zero-sum shift in popularity between golf types but rather as an expansion and diversification of the broader leisure sports industry.

Fifth, loyalty was found to be significantly higher in the park golf group than in both the field and VR golf groups. This reflects the remarkable popularity and rapid growth of park golf in recent years (Yeo & Kwon, 2024). High loyalty indicates that park golf effectively satisfies participants’ physical, psychological, and social needs. In contrast, the relatively lower loyalty observed among field and VR golf participants corresponds to their higher switching intention (field golf) and the declining participation trend among the MZ generation (VR golf; Yun & Seo, 2024). These findings suggest that both field and VR golf face potential challenges in maintaining and securing a loyal customer base. It may, therefore, be necessary for these traditional and technology-based formats to develop strategies that stimulate interest among leisure participants with diverse motivations. In addition, this trend may also be partly attributed to the natural demographic shift driven by population aging.

Sixth, continuous participation intention showed patterns consistent with the key variables of loyalty and switching intention. The park golf group exhibited the highest level of continuous participation intention, exceeding that of the other two groups by a considerable margin. This is particularly meaningful given that park golf remains a relatively new sport. Such strong intention to continue participation demonstrates that park golf has transcended the level of a temporary fad and has established itself as a sustainable outdoor leisure activity that meets the growing needs of an aging society (S.-H. Kim et al., 2025). The previously identified high levels of physical and psychological satisfaction are likely the key driving factors behind this strong commitment. Conversely, the relatively low continuous participation intention observed among field golf participants underscores the urgent need for strategies that reduce participation costs and attract new demographics to sustain the traditional golf market.

From a practical perspective, the results suggest that the sustainability of the golf industry hinges on segmented management. While park golf demonstrates high loyalty among older adults, field golf operators must address cost and time constraints to mitigate participant attrition. Implementing flexible pricing models is a critical measure to alleviate the economic burdens currently driving market exit.

From an academic standpoint, the strength of this study lies in its integrated framework, which compares psychological and behavioral characteristics across three distinct golf sub-types within a single analytical model. However, several limitations regarding generalizability must be addressed. Although the sample size was sufficient for multivariate analysis, the use of non-probability convenience sampling primarily within urban areas may limit the national representativeness of the findings. Thus, caution is warranted when generalizing these results to the broader population, and future research should employ stratified sampling techniques to enhance external validity.

## 6. Conclusions

This study empirically identifies the psychological and behavioral shifts occurring as the Korean golf market diversifies into field, VR, and park golf. The findings underscore that long-term industry sustainability depends on the development of type-specific management strategies that effectively align with the evolving needs and constraints of different generational segments.

## Figures and Tables

**Table 1:** Results of validity and reliability.

Construct	Items	λ	S.E.	C.R.	Cronbach’s α	CR	AVE
Physical Satisfaction	Helping me improve my physical fitness	0.89	-	-	0.851	0.855	0.664
	Helping me recover physically	0.82	0.042	19.52 ***			
	Helping me maintain my health	0.78	0.048	16.25 ***			
Mental Satisfaction	Being interesting to me	0.88	-	-	0.841	0.846	0.648
	Giving me confidence	0.81	0.045	18.00 ***			
	Giving me a sense of accomplishment	0.75	0.051	14.71 ***			
Social Satisfaction	Helping me interact socially with others	0.91	-	-	0.837	0.84	0.639
	Helping me build close relationships	0.82	0.044	18.64 ***			
	Spending my free time with other participants	0.76	0.049	15.51 ***			
Switching Intention	No intention to switch golf types	0.90	-	-	0.879	0.882	0.715
	No intention to change physical activities	0.85	0.038	22.37 ***			
	Not choosing another activity	0.81	0.041	19.76 ***			
Loyalty	Continuing my current golf type	0.86	-	-	0.858	0.864	0.614
	Frequent participation	0.82	0.043	19.07 ***			
	Recommending to others	0.78	0.047	16.60 ***			
	Engaging with related online communities	0.74	0.052	14.23 ***			
Continuous Participation	Continuing participation in the future	0.84	-	-	0.806	0.815	0.528
	Choosing it over alternatives	0.79	0.046	17.17 ***			
	Participating despite burdens	0.74	0.053	13.96 ***			
	Participating again if possible	0.72	0.055	13.09 ***			

Note. *** *p* < 0.001.

**Table 2:** Descriptive statistics of survey respondents.

	Sub-Categories	Group 1	Group 2	Group 3
		Field Golf	VR Golf	Park Golf
Gender	Male	52 (53.1%)	78 (59.1%)	56 (57.7%)
	Female	46 (46.9%)	54 (40.9%)	41 (42.3%)
Age	20s	18 (18.4%)	24 (18.2%)	10 (10.3%)
	30s	27 (27.6%)	31 (23.5%)	18 (18.6%)
	40s	35 (35.7%)	57 (43.2%)	35 (36.1%)
	50s	14 (14.3%)	12 (9.1%)	12 (12.4%)
	Above 60 years	4 (4.1%)	8 (6.1%)	22 (22.7%)
Golf experience (years)	Less than 1 year	29 (29.6%)	42 (31.8%)	19 (19.6%)
	1 to <3 years	50 (51.0%)	62 (47.0%)	31 (32.0%)
	3 to <5 years	16 (16.3%)	22 (16.7%)	21 (21.6%)
	Over 5 years	3 (3.1%)	6 (4.5%)	26 (26.8%)
Participation time per week	Less than an hour	5 (5.1%)	12 (9.1%)	17 (17.5%)
	1–3 h	30 (30.6%)	11 (8.3%)	5 (5.2%)
	3–5 h	57 (58.2%)	83 (62.9%)	75 (77.3%)
	More than 5 h	6 (6.1%)	26 (19.7%)	-
Total		98 (100.0%)	132 (100/0%)	97 (100/0%)

**Table 3:** Results of MANOVA.

Variables	Sub-Factors	*df*	*F*	*p*	*η* ^2^	*post hoc*		Mean	
							G1	G2	G3
Participation Satisfaction	Physical	2	5.497	0.004 **	0.033	b < c	3.432	3.258	3.588
	Mental	2	4.475	0.012 *	0.027	a,b < c	3.391	3.419	3.680
	Social	2	1.422	0.243	0.009	-	3.684	3.568	3.505
Switching intention		2	15.566	0.001 ***	0.088	a > b,c	3.680	3.278	3.007
Loyalty		2	14.416	0.001 ***	0.080	a,b < c	3.207	3.303	3.735
Continuous participation intention		2	18.797	0.001 ***	0.104	a,b < c	3.554	3.593	4.072

Note. a = field, b = VR, c = park; *** *p* < 0.001, ** *p* < 0.01, * *p* < 0.05.

**Table 4:** Results of post hoc analyses.

		Participation Satisfaction			Switching Intention	Loyalty	Continuous Participation Intention
		Physical	Mental	Social			
Field	VR	0.220	0.962	0.516	0.002 **	0.626	0.908
	Park	0.350	0.030 *	0.255	0.001 ***	0.001 ***	0.001 ***
VR	Field	0.220	0.962	0.516	0.002 **	0.626	0.908
	Park	0.005**	0.037 *	0.822	0.060	0.001 ***	0.001 ***
Park	Field	0.350	0.030 *	0.255	0.001 ***	0.001 ***	0.001 ***
	VR	0.005**	0.037 *	0.822	0.060	0.001 ***	0.001 ***

Note. *** *p* < 0.001, ** *p* < 0.01, * *p* < 0.05.

## Data Availability

The original contributions presented in the study are included in the article; further inquiries can be directed to the corresponding author.
